# Internal noise measures in coarse and fine motion direction discrimination tasks and the correlation with autism traits

**DOI:** 10.1167/jov.22.10.19

**Published:** 2022-09-23

**Authors:** Edwina R. Orchard, Steven C. Dakin, Jeroen J. A. van Boxtel

**Affiliations:** 1 Department of Psychology, Faculty of Arts and Sciences, Yale University, New Haven, CT, USA; 2 Yale Child Study Center, School of Medicine, Yale University, New Haven, CT, USA; 3 School of Optometry & Vision Science, University of Auckland, Auckland, New Zealand; 4 New Zealand National Eye Centre, University of Auckland, Auckland, New Zealand; 5 UCL Institute of Ophthalmology, University College London, London, UK; 6 Discipline of Psychology, Faculty of Health, University of Canberra, Bruce, ACT, Australia; 7 Turner Institute for Brain and Mental Health, School of Psychological Sciences, Monash University, Clayton, Australia

**Keywords:** autism, motion perception, internal noise, double-pass

## Abstract

Motion perception is essential for visual guidance of behavior and is known to be limited by both internal additive noise (i.e., a constant level of random fluctuations in neural activity independent of the stimulus) and motion pooling (global integration of local motion signals across space). People with autism spectrum disorder (ASD) display abnormalities in motion processing, which have been linked to both elevated noise and abnormal pooling. However, to date, the impact of a third limit—induced internal noise (internal noise that scales up with increases in external stimulus noise)—has not been investigated in motion perception of any group. Here, we describe an extension on the double-pass paradigm to quantify additive noise and induced noise in a motion paradigm. We also introduce a new way to experimentally estimate motion pooling. We measured the impact of induced noise on direction discrimination, which we ascribe to fluctuations in decision-related variables. Our results are suggestive of higher internal noise in individuals with high ASD traits only on coarse but not fine motion direction discrimination tasks. However, we report no significant correlations between autism traits and additive noise, induced noise, or motion pooling in either task. We conclude that, under some conditions, the internal noise may be higher in individuals with pronounced ASD traits and that the assessment of induced internal noise is a useful way of exploring decision-related limits on motion perception, irrespective of ASD traits.

## Introduction

Although deficits in social, behavioral, and cognitive functioning form the core symptomology of autism spectrum disorder (ASD), sensory and perceptual abnormalities have long been associated with the condition (Asperger, 1944; Grandin, 1992; Kanner, 1943; Kern et al., 2006; O'Neill & Jones, 1997). Sensory issues likely contribute to the complex pattern of behaviors that define ASD, as they are evident in social deficits (facial perception, gestural interpretation, unusual eye contact, difficulties with joint attention) and non-social deficits (light sensitivity, repetitive/stereotyped behaviors) (Simmons, Robertson, McKay, Toal, McAleer, & Pollick, 2009). Differences in sensory processing may play a causative role in core features of autism (Marco, Hinkley, Hill, & Nagarajan, 2011), such as language delay (auditory processing) and difficulty with reading emotion from faces (visual processing). Understanding the mechanisms of such sensory deficits may therefore help to reveal the neural underpinnings of ASD (Zwaigenbaum, Bryson, Rogers, Roberts, Brian, & Szatmari, 2005).

The etiology of sensory abnormalities in ASD is unknown, but recent work has suggested that higher levels of variability in neural response (internal noise) could be a physiological basis for the condition. Brain imaging studies have shown that individuals with ASD have increased internal noise (Dinstein, Heeger, Lorenzi, Minshew, Malach, & Behrmann, 2012; Domínguez, Velázquez, & Galán, 2013; Milne, 2011; Weinger, Zemon, Soorya, & Gordon, 2014). Specifically, the variability (but not magnitude) of evoked functional magnetic resonance imaging (fMRI) response was larger in people with ASD, so that signal-to-noise ratios were lower across visual, auditory, and somatosensory cortices (Dinstein et al., 2012). Similar differences in neural variability have been reported using resting-state magnetoencephalography (Domínguez et al., 2013), suggesting that high internal noise may represent a fundamental physiological difference in cortical processing of people with ASD (but see Butler, Molholm, Andrade, & Foxe, 2017; Coskun et al., 2009).

At a behavioral level, the impact of internal noise on visual perception in ASD has mostly been investigated in the motion and orientation domain (Manning, Tibber, Charman, Dakin, & Pellicano, 2015; Manning, Tibber, & Dakin, 2017; Park, Schauder, Zhang, Bennetto, & Tadin, 2017; Zaidel, Goin-Kochel, & Angelaki, 2015). Early evidence for motion processing deficits in ASD indicated that people with ASD were significantly poorer at reporting the perceived direction of stimuli defined by contrast change than controls but showed no differences with stimuli defined by luminance change (Bertone, Mottron, Jelenic, & Faubert, 2003).

Later research has used motion coherence tasks (Simmons et al., 2009) to measure the minimum number of coherently moving dots (i.e., in a common direction) within a population of randomly moving dots required to support a reliable report of direction. This work has shown higher motion coherence thresholds in ASD compared with controls (Milne, Swettenham, Hansen, Campbell, Jeffries, & Plaisted, 2002; Spencer & O'Brien, 2006), although not consistently (Brieber, Herpertz-Dahlmann, Fink, Kamp-Becker, Remschmidt, & Konrad, 2010; Jones et al., 2011; Manning et al., 2015). Overall, ASD groups exhibit more variable levels of performance compared with controls, speaking to variability within ASD in general (Milne, White, Campbell, Swettenham, Hansen, & Ramus, 2006; Pellicano, Gibson, Maybery, Durkin, & Badcock, 2005). Although behavioral data are equivocal, there is evidence that, even when behavioral impairments are absent, neural differences exist between individuals with and without ASD (Brieber et al., 2010; Freitag et al., 2008; Herrington et al., 2007; Kaiser et al., 2010; McKay, Simmons, McAleer, Marjoram, Piggot, & Pollick, 2012; Peiker et al., 2015). Could internal noise contribute to such processing differences?

There are two broad explanations for atypical motion coherence thresholds in ASD. First, coherence thresholds could be increased due to poor estimation of local direction due to high levels of internal noise (Barlow & Tripathy, 1997; Zaidel et al., 2015). Second, coherence thresholds could be increased due to impaired motion pooling (i.e., integration) of local direction signals (Dakin, Mareschal, & Bex, 2005a). Pooling local motion signals would combat external (and internal) noise on local motion signals so that a deficit in this process would degrade direction estimation (Dakin et al., 2005a; Manning et al., 2015). A recent study investigated these processes in ASD (Manning et al., 2015) and found no evidence for a difference in internal noise between the two groups. However, they did find a difference in motion pooling. Contrary to expectation, individuals with ASD showed more motion pooling when external noise was high, causing ASD children to outperform controls. This finding, however, was not confirmed in a subsequent study, although combining both studies still showed significantly more motion pooling (Manning et al., 2017). These results are consistent with findings of enhanced motion perception in ASD compared to controls using a different experimental design (Foss-Feig, Tadin, Schauder, & Cascio, 2013).

**Figure 1.**
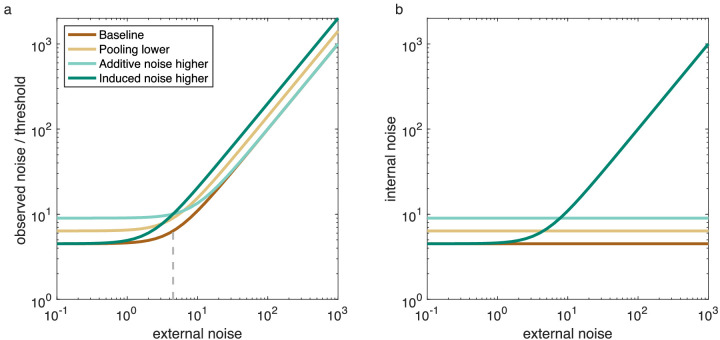
(a) Modeled threshold versus external noise curves, as measured with equivalent noise paradigms. As external (stimulus) noise is increased, performance thresholds (i.e., observed noise) rise. When compared to a baseline (with a certain level of additive but no induced noise), the following effects are observed: Reducing motion pooling leads to a uniform upward shift in the curve; increasing additive noise increases thresholds but only at low levels of external noise; and induced noise increases thresholds, especially at high levels of external noise. The vertical dashed line identifies the elbow in the baseline curve and quantifies the level of additive noise according to the equivalent noise paradigm. (b) Internal noise versus external noise curves, as obtained with a double-pass paradigm. When expressed in terms of the total amount of internal noise, there are clear differences between the different manipulations. Increasing additive noise elevates the internal noise by the same amount independent of the external noise. When induced noise is included, internal noise shows a strong dependence on external noise. Induced noise is the only variable that causes an increase in internal noise in the internal versus external noise plot. In the original approach by Burgess and Colborne (1988), motion pooling was not taken into account. When using this approach, motion pooling scales the curve up or down equally at all external noise levels (causing misestimated noise levels, as shown in this figure). However, when explicitly including motion pooling in the model, motion pooling would have no effect on internal noise estimates (as per our method).

The equivocal nature of the literature suggests that internal additive noise may not be a strong determinant of motion processing in ASD. However, the ASD literature to date has largely ignored the potential interaction between external noise and internal noise (but see a recent exception, Park et al., 2017, looking at orientation perception). Internal noise can be divided into at least two components: additive and induced (Burgess & Colborne, 1988; Lu & Dosher, 2008). Additive noise is the internal “baseline” level of noise, which is constant across different amounts of input. It is this type of noise that is measured by previous paradigms (i.e., equivalent noise paradigms) (Dakin et al., 2005a; Lu & Dosher, 2008). Induced noise, on the other hand, is proportional to the amount of noise present in the stimulus (Burgess & Colborne, 1988).^1^ Importantly, when external noise is low (and thus induced noise is low), the main source of internal noise is additive noise. As external noise increases, induced noise increases also and becomes the main source of internal noise. Figure 1a shows the impact of different types of noise and pooling on thresholds (as measured, for example, using equivalent noise paradigms). As is clear from this figure, it is difficult to distinguish between the different types of noise. To a large degree (but not completely), the changes in different types of noise are interchangeable; for example, additive noise and induced noise together may look like a change in motion pooling. It is thus possible that previous studies of motion perception using equivalent noise paradigms, for example, failed to identify changes in induced noise because they were interpreted as a change in motion pooling and additive noise.

In this study we circumvent this problem by obtaining measures of additive noise, induced noise, and pooling using the double-pass paradigm (Burgess & Colborne, 1988), which is considered to be a direct way of estimating internal noise (Burgess & Colborne, 1988; Lu & Dosher, 2008). In the double-pass paradigm, identical noisy stimuli are presented twice to individuals. A person with high internal noise might perceive such identical stimuli as different (Green, 1964; Haigh, Heeger, Dinstein, Minshew, & Behrmann, 2015), but, because the stimuli are identical on both presentations, any perceptual difference must be due to internal processes (Green, 1964). The advantage of the double-pass paradigm is that it does not assume that all internal noise is additive and allows one to distinguish the impact of induced noise from other types of noise (Burgess & Colborne, 1988; Lu & Dosher, 2008) (Figure 1b). The method has previously been employed to measure internal noise correlations with autism traits in the typically developing population on three non-motion tasks (Vilidaite, Yu, & Baker, 2017), although no estimates of induced noise were derived.

Because the double-pass paradigm has not been used for motion discrimination, we here further develop it to deal with circular variables. We further tested the influence of additive and induced noise in both a coarse and a fine motion direction discrimination task, because these tasks may depend on different sensory decoding rules (Jazayeri & Movshon, 2007) and thus may be differently affected by noise. The difference between fine and coarse motion judgments has not been previously investigated in the context of ASD. However, as ASD is often linked to a more detail-oriented processing, comparing fine and coarse discrimination tasks is potentially very revealing with regard to the underlying mechanisms that are affected in motion processing in ASD.

## Model development

The induced noise model has been extensively described previously (Burgess & Colborne, 1988; Lu & Dosher, 2008). Here, we briefly recapitulate the features and assumptions of the model and describe details of our version of the model. The model is *descriptive*, setting out to determine the relative contributions of different types of noise to performance. The model assumes three independent normally distributed types of noise that contribute to the overall noise (i.e., their variances add). These types are additive noise (σ_add_), external stimulus noise (σ_ext_), and induced noise (σ_ind_). Their combined impact is determined by adding their variances:

(1) $$\sigma_{\mathrm{total}}^{2}=\sigma_{\mathrm{add}}^{2}+\sigma_{\mathrm{ind}}^{2}+\sigma_{\mathrm{ext}}^{2}$$

where σ_total_ is the total noise that determines the sensitivity of the system (e.g., detection threshold). Both additive noise and induced noise are internal to the brain. Additive noise describes the combination of all noises that together are independent of the level of external noise. The induced noise describes all noise components that together are proportional to the external noise (Burgess & Colborne, 1988). There are no assumptions about the order in which these sources are added^2^.

Because σ_ind_ is a proportion (*m*) of external noise (σ_ind_ = *m*σ_ext_), this can be rewritten as

(2) $$\begin{array}{ccc} \sigma_{\mathrm{total}}^{2}\; & = & \sigma_{\mathrm{add}}^{2}+{\left(m\sigma_{\mathrm{ext}}\right)}^{2}+\sigma_{\mathrm{ext}}^{2}\\ \; & = & \sigma_{\mathrm{add}}^{2}+\left(1+m^{2}\right)\sigma_{\mathrm{ext}}^{2} \end{array}$$

Performance often differs from this optimal combination by a simple scaling factor, and conventionally a constant denoting efficiency, or a perceptual template gain (γ), is included (Burgess & Colborne, 1988; Lu & Dosher, 2008):

(3) $$\sigma_{\mathrm{total}}=\frac{1}{\gamma }\sqrt{\sigma_{\mathrm{add}}^{2}+\left(1+m^{2}\right)\sigma_{\mathrm{ext}}^{2}}$$

In the current manuscript, we use a mathematically equivalent form, where a constant *n* is interpreted as motion pooling (Dakin, Mareschal, & Bex, 2005a; van Boxtel, 2019):

(4) $$\sigma_{\mathrm{total}}=\sqrt{\frac{\sigma_{\mathrm{add}}^{2}+\left(1+m^{2}\right)\sigma_{\mathrm{ext}}^{2}}{n}}$$

This formulation is similar to the central limit theorem, with *n* describing the number of samples that are taken to calculate the mean. The model assumes independence of noise, linear addition of their variances, and a linear and optimal detection process. In our model, we do not include an assumption of noisy motion pooling. This is because simulations showed that assuming noisy motion pooling (i.e., pooling over different numbers of moving dots on different trials) does not materially affect internal noise estimates (see Simulations in https://osf.io/4gdkt/). Increasing noise in the motion pooling increases estimates of average motion pooling, suggesting that it is not possible to separate average motion pooling from variation in motion pooling.

## Experiment 1. Coarse motion direction discrimination

In the first experiment, we investigated the dependence of motion direction discrimination thresholds on additive and induced noise in a coarse direction discrimination task. We used the double-pass paradigm to estimate both sources of noise and develop an experimental approach to estimation of motion pooling.

### Method

#### Participants

Ethics approval was obtained from the Monash University Human Research Ethics Committee, and written informed consent was obtained from all participants prior to participation. This study was completed in accordance with approved guidelines. Participants—45 healthy adults (31 female, 14 male), ranging in age from 18 to 40 years old (*M*_age_ = 22.07, *SD*_age_ = 4.96)—were recruited from the Monash University Clayton campus. All participants were proficient in English and had normal or corrected-to-normal vision, fulfilling our inclusion criteria. Participants received monetary compensation for their participation. Participants were excluded if the internal noise model (explained below; Equation 5 fitted with an *R*^2^ < 0.5). One participant was excluded based on this exclusion criterion.

#### Materials

The Autism Spectrum Questionnaire (AQ) was used to measure self-reported autistic traits (Baron-Cohen, Wheelwright, Skinner, Martin, & Clubley, 2001). The AQ is comprised of 50 items on a four-point Likert scale (definitely agree, slightly agree, slightly disagree, definitely disagree) and was administered and scored on a computer.

We also collected data from the Kaufman Brief Intelligence Test Second Edition (KBIT-2) (Kaufman & Kaufman, 2004). Because our cohort included a large proportion of non-native English speakers, this test did not provide accurate estimates of the intelligence quotient, but all individuals scored >70.

#### Apparatus

This study was completed in an experimental room without external lights. Artificial lights were turned on, which we expect had no impact on our results because stimuli were displayed far above detection threshold. Participants sat comfortably in front of the computer, with their head stabilized by a chin rest. The experiment was displayed on a VIEWPixx/3D monitor (VPixx Technologies, Saint-Bruno-de-Montarville, Canada), which had a refresh rate of 120 Hz and a resolution of 1920 × 1200 pixels and was viewed from a distance of 114 cm. All experimental displays (including stimuli and AQ administration and scoring) were created using MATLAB (MathWorks, Natick, MA) and OpenGL with Psychtoolbox extensions (Brainard, 1997; Pelli, 1997).

#### Stimuli

**Figure 2.**
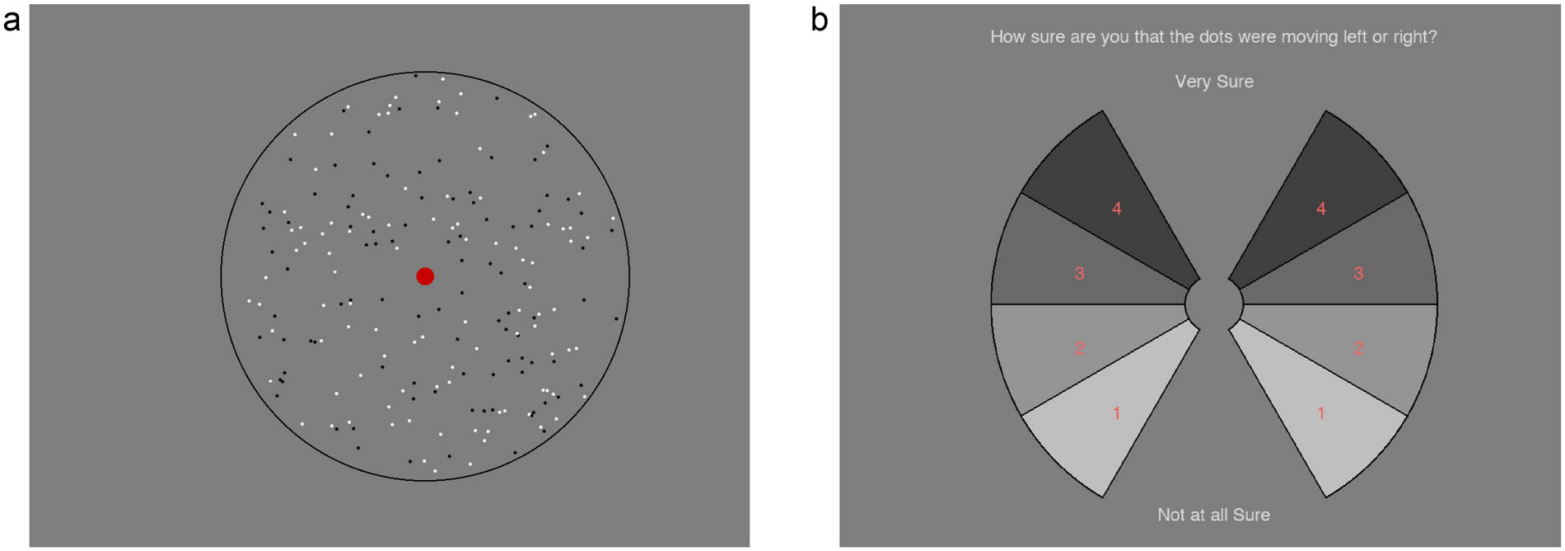
Screenshot of visual motion task stimuli (a) and response screen (b). Participants viewed the stimuli and then gave a response by clicking on a wedge in the response screen indicating whether the dots were moving clockwise or anticlockwise of vertical and how confident they were in their response.

We used random-dot motion stimuli (Figure 2), made up of 200 circular dots (100 black, 100 white; diameter, 0.07°) moving within a circular aperture, which was outlined in black against a gray background. A red fixation mark was provided (diameter, 0.35°), which was surrounded by a 0.67° exclusion zone in which no dots were drawn in order to decrease potential pursuit eye movements. Each individual dot moved (speed = 2.38°/s; lifetime = 8 frames) in a direction randomly chosen from a wrapped normal Gaussian distribution (mean 22° clockwise or anticlockwise from vertical, with standard deviations of 0°, 35°, 45°, 60°, 70°, 80°, 90°, or 100°). A noise level of zero means that all dots moved in the same direction (22° clockwise or anticlockwise of upward motion).

Participants were required to indicate the perceived direction of the motion stimuli using a response screen (Figure 2b). This consisted of a confidence wheel comprised of eight numbered wedges. The four wedges on the left or right were used to indicate that the dots were, on average, perceived to move anticlockwise or clockwise of vertical, respectively. Each of the wedges was numbered (1 = not at all sure, 4 = very sure) and shaded (1 = lightest, 4 = darkest), and the confidence screen was flipped upside down every 100 trials.

#### Procedure

Participants performed the KBIT-2, which took approximately 40 minutes, then the visual motion task, and finally the AQ questionnaire.

In the motion task, stimuli (Figure 2a) were presented for 0.75 seconds followed by the response screen (Figure 2b). Participants were instructed to focus their gaze on the fixation point (red dot) at the center of the screen during stimulus presentation and were asked to judge the average direction of the moving dots. Participants indicated their decision by a clicking on a wedge in the response screen which served to indicate if they thought dots were moving clockwise or anticlockwise of vertical and how confident they were in this judgment (Figure 2b).

Our study employed a double-pass paradigm, similar to that used by Burgess and Colborne (1988), which is a method whereby two identical presentations (passes) of each stimulus are made over two separate trials. There were 100 unique stimuli and therefore 200 trials in total for each noise level. The first passes were run first in random order, and the second passes were then run in the same order. Participants were given self-timed breaks after every 100 trials. A total of 1600 trials were run.

### Data Analysis

Analyses were run in MATLAB, and Bayesian statistics were performed using the jsq module from JASP run in jamovi (jamovi, 2020; JASP Team, 2018).

We employed a double-pass paradigm to quantify internal noise (Burgess & Colborne, 1988). Values for internal additive and induced noise and motion pooling were calculated based on participants’ performance on the visual motion task. Internal noise can be estimated by examining the accuracy (over individual trials) and consistency (between the two passes) of a person's response (Burgess & Colborne, 1988). For this task, a person is accurate if the dots are moving left (or right) and the participant responds “left” (or “right”). A person is consistent if they choose the same direction on both test and retest presentations of a trial, regardless of whether or not they were correct (and regardless of their reported confidence).

#### Internal noise

**Figure 3.**
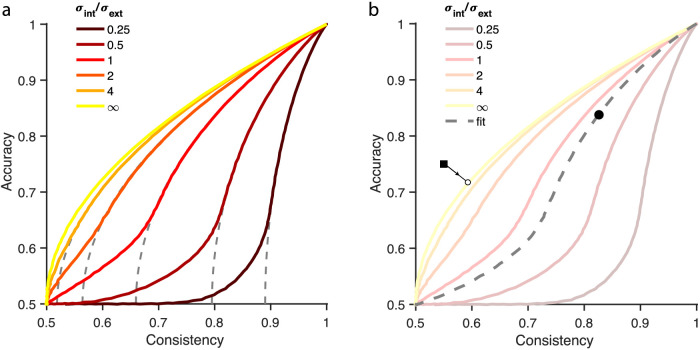
Relationship between accuracy and consistency, dependent on different ratios of  σ_int_/σ_ext_. (a) Colored lines were calculated using a wrapped Gaussian distribution, and the dashed gray lines are based on Gaussian distributions used by Burgess and Colborne (1988). The line for σ_int_/σ_ext_ = ∞ is the case without external noise. (b) Schematic of how data were analyzed for two example data points. Internal noise for the data falling above the  σ_int_/σ_ext_ = ∞ curve (e.g., square) was estimated by finding the nearest point on the σ_int_/σ_ext_ = ∞ curve, and the internal noise associated with that point was taken as the measured internal noise. For all other data (circle), the best fitting line determined σ_int_/σ_ext_.

When noise levels are expressed as a ratio of internal over external noise (σ_int_/σ_ext_), there exists a fixed (nonlinear) relationship between accuracy and consistency at different levels of σ_int_/σ_ext_ (Burgess & Colborne, 1988). These relationships are shown in Figure 3a for different ratios of σ_int_/σ_ext_ (gray dashed lines). Internal noise values can be calculated by fitting such a curve to experimental data and finding the ratio σ_int_/σ_ext_ that best captures the data. Then, because σ_ext_ is known, σ_int_ can be calculated. Because this approach will not work for circular variables, we calculated the curves numerically for wrapped circular distributions. Figure 3a shows a comparison of the noncircular (colored lines) and circular (gray dashed lines) approaches, using identical parameters. When noise is large (toward the lower side in the plot), circular data deviate from the noncircular data. The curving toward the point where accuracy and consistency are both 50% occurs because, when external noise levels are large, dots at the extreme end of the distribution will be wrapped around the circle (or more than once). Some information remains as long as internal noise is not heavily wrapped around, but because it is a proportion of external noise it will also wrap around at large values of external noise. The distribution will approach a uniform distribution on a circle. At that point, the stimulus is largely uninformative, and both accuracy and consistency are random. An additional unfortunate consequence of the circular nature of the data is that the exact shape of the curves is dependent on the signal strength. When the mean angle (i.e., signal) is small, the data are well approximated by an approach using Gaussian distributions (only showing deviations at very large noise values).

Instead of fitting the data, we constructed a large lookup table for accuracy-versus-consistency curve ratios σ_int_/σ_ext_. We used an ad hoc function to achieve a reasonable coverage of ratios: (1.12*^q^*–1)/25, with *q* ranging from 1 to 59 in steps of 0.5. Individual data points were compared with these curves, and the curve to which the data point showed the smallest squared Euclidian distance was taken as the best fit (Figure 3b). The squared distance was calculated taking into account deviations in terms of both accuracy and consistency. When the participant's data showed an accuracy below 50% it was adjusted to (1-accuracy). This happened six times (out of 45 × 8 = 360 data points, or 1.67% of all data points). Note that this correction does not materially change the data, because the consistency–accuracy curves are mirror symmetric along the line accuracy = 50%. However, keeping all points above 50% accuracy simplifies the fitting.

This procedure determined σ_int_/σ_ext_, as well as σ_int_ (as σ_ext_ is known). In some cases, data points fell above the curve σ_int_/σ_ext_ = ∞ (e.g., square in Figure 3b). As can be seen from Figure 3b, the data cannot be fit with a theoretical curve. Because we have a limited amount of trials per participant, some points in our dataset fell in this region. The fact that the data lie in that region indicates that the data was dominated by internal noise. Therefore, for those points, internal noise was estimated by fitting the σ_int_/σ_ext_ = ∞ curve to the data; that is, we found the point on this curve that was closest to the data point (square in Figure 3b), where the squared error (in both consistency and accuracy directions) was smallest. The internal noise value that was associated with that point on the curve was taken as the internal noise estimate for our data point. Simulations showed that this resulted in correct approximations of internal noise levels (see Simulations in https://osf.io/4gdkt/).

#### Determining additive and induced internal noise

In equivalent noise paradigms, the amount of internal (additive) noise is determined by finding the elbow in the curve in Figure 1a (dashed line). This approach, however, ignores the possible contributions of induced noise. Therefore, if induced noise influences the task at hand, then estimates of additive noise can be incorrect. For example, when introducing induced noise in the data of Figure 1a, the elbow moves to lower external noise values, thus underestimating the amount of additive noise. Unfortunately, it is difficult to determine from the plots in Figure 1a whether induced noise is present, because the curves are very similar (and can be made nearly overlapping by appropriately setting additive noise and pooling parameters). However, using the double-pass paradigm, one can plot the internal noise versus external noise values (Figure 1b). Induced noise can be derived from the increase in internal noise that depends on the level of external noise, with a slope of 0 indicating that there is no significant influence of induced noise. This makes intuitive sense, as the internal noise consists only of a fixed level of constant additive noise, which is not changed by the level of external noise, thus tracing a flat line in the internal versus external noise plot.

As indicated, according to Burgess and Colborne (1988), the induced noise σ_ind_ is directly proportional to the external noise; thus, σ_ind_ = *m*σ_ext_. The total internal noise is σ_int_ = sqrt(σ_add_^2^ + σ_ind_^2^) = sqrt(σ_add_^2^ + *m*^2^σ_ext_^2^).

The induced noise factor *m* can be derived in various ways, and, because we had no a priori reason to assume which one worked best, we performed a simulation study (see the Appendix). This simulation study showed that the best way to determine additive and induced noise was to fit the following function to the collective internal noise data per participant:

(5) $$f\left(\sigma_{\mathrm{ext}}\right)=\sqrt{\frac{\sigma_{\mathrm{add}}^{2}+{\left(m\;\sigma_{\mathrm{ext}}\right)}^{2}}{n}}$$

where *n* is the number of motion samples that are taken to estimate motion direction (i.e., motion pooling). We fitted Equation 5 to individual participant data after first independently estimating *n* from the data (see below).

#### Determining motion pooling

**Figure 4.**
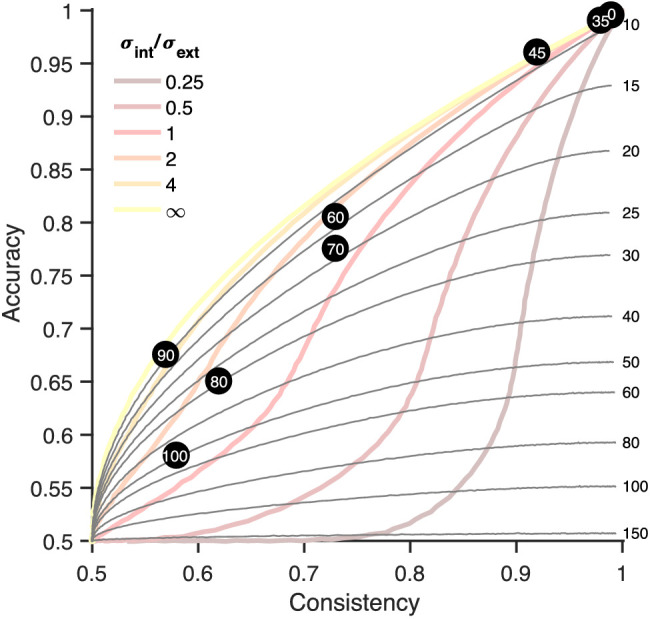
Observed external noise and motion pooling. This plot has the same layout as Figure 3 but also shows iso-external noise lines: the accuracy and consistency expected for a given external noise level without motion pooling (displayed on the right). The black dots are experimental data for one participant, and the external noise for each condition is indicated within the dot. The external noise for each condition is higher than the iso-external noise line on which it falls, suggesting that the observed/effective external noise was lower than the actual external noise, an indication that motion pooling was occurring.

Better performance in a task may result not only from lower noise levels but also when the direction information from multiple dots is combined. This is called (global) motion pooling (Dakin et al., 2005a). To derive motion pooling in our task, we plotted iso-external noise lines (Figure 4). These lines trace, for one value of external noise, the expected values of consistency and accuracy without motion pooling. Note that these lines are dependent on the parameters of our stimulus and thus will be different in different experiments, such as the fine discrimination task below. Each data point will fall on only one curve, and this curve indicates the *observed* external noise. The data generally fall on a curve that has a lower level of observed external noise than the amount of external noise that was actually present in the stimulus, indicating that information from multiple dots is pooled (see, for example, the data points from σ_ext_ = 80, which falls on the line of observed external noise = 30) (Figure 4).

The level of motion pooling is derived with a characteristic of the central limit theorem that

(6) $$\sigma_{\mathrm{obs}}=\;\sqrt{\frac{\sigma_{\mathrm{int}}^{2}+\sigma_{\mathrm{ext}}^{2}}{n_{\mathrm{samp}}}}$$

where σ_obs_ is the observed standard deviation of the response, and *n*_samp_ is the effective number of samples that are combined to give a motion direction estimate (i.e., motion pooling). In our case,

(7) $$\sigma_{\mathrm{obs}}=\;\sqrt{\frac{\sigma_{\mathrm{int}}^{2}+\sigma_{\mathrm{ext}}^{2}}{n_{\mathrm{samp}}}}=\sqrt{s_{\mathrm{int}}^{2}+s_{\mathrm{ext}}^{2}}$$

where σ_int_ and σ_ext_ are the real internal and external noise values, and *s*_int_ and *s*_ext_ are observed values. The observed values have the motion sampling already taken into account, which is why *n*_samp_ does not appear on the right-hand side of the equation. Because we know the ratio (α) between internal and external noise and thus σ_int_ = α * σ_ext_, this equation can be rewritten as

(8) $$\sqrt{\alpha_{1}s_{\mathrm{ext}}^{2}+s_{\mathrm{ext}}^{2}}=\sqrt{\frac{\alpha_{2}\sigma_{\mathrm{ext}}^{2}+\sigma_{\mathrm{ext}}^{2}}{n_{\mathrm{samp}}}}$$

and, rewriting, we obtain

(9) $$\sqrt{\left(1+\alpha_{1}\right)s_{\mathrm{ext}}^{2}}=\sqrt{\frac{\left(1+\alpha_{2}\right)\sigma_{\mathrm{ext}}^{2}}{n_{\mathrm{samp}}}}$$

which can be rewritten as

(10) $$n_{\mathrm{samp}}=\frac{\left(1+\alpha_{2}\right)\sigma_{\mathrm{ext}}^{2}}{\left(1+\alpha_{1}\right)s_{\mathrm{ext}}^{2}}$$

Assuming that α_1_ = α_2_ (i.e., that the motion pooling does not affect the ratio of internal to external noise), we can derive motion pooling as

(11) $$n_{\mathrm{samp}}=\frac{\sigma_{\mathrm{ext}}^{2}}{s_{\mathrm{ext}}^{2}}$$

Because we know both σ_ext_ (the external noise, or standard deviation of directional noise) and *s*_ext_ (the observed external noise), we can calculate the motion pooling, *n*_samp_. Simulations showed that this method of estimation works but only for the lower noise levels (we used 35°, 45°, and 60°) for our coarse discrimination task and for the higher noise levels for the fine discrimination task (3.71–51.19). These simulations can be found in Simulations at https://osf.io/4gdkt/.

For data points that lay beyond the σ_int_/σ_ext_ = ∞ curve, observed external noise could not be determined, and these data points were consequently not used to estimate motion pooling.

### Results

#### Influence of external noise on accuracy, consistency, and confidence

**Figure 5.**
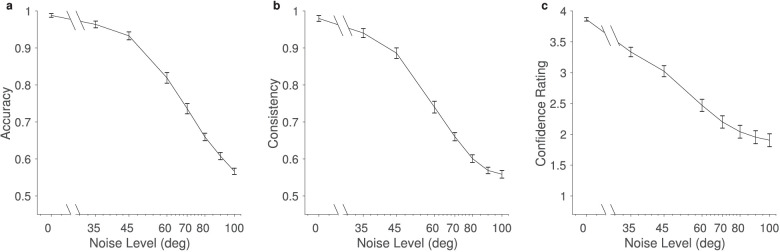
Mean behavioral performance. (a) Accuracy, (b) consistency, and (c) confidence decreased as external noise level increased but remained significantly above chance level at all noise levels. Error bars are standard errors of the mean calculated over participants.

As external noise increased, accuracy, consistency, and confidence ratings decreased, reflecting increased task difficulty (Figure 5). In particular, both accuracy and consistency decreased from near-perfect to near-chance levels. This suggests that the spread of noise levels measured both the upper and lower limits of participant performance. Although we do not present any further analysis of confidence ratings here, confidence data have been deposited in a large, openly available confidence database (Rahnev et al., 2020).

We investigated whether AQ had a significant effect on the dependence of accuracy and consistency on noise. Numerically, the accuracy and consistency were lower in people with higher AQ scores. To test the statistical significance of this finding, we constructed an unrestricted linear model with AQ, noise level, and their interaction as terms, and we compared them to models without the interaction and models without both the interaction and the AQ term. Likelihood ratio tests revealed no significant differences between unrestricted models and those excluding the interaction [accuracy: χ^2^(1) = 0.07, *p* = 0.78; consistency: χ^2^(1) = 0.68, *p* = 0.41] or between unrestricted models and those excluding both AQ and the interaction [accuracy: χ^2^(2) = 3.51, *p* = 0.17; consistency: χ^2^(2) = 4.08, *p* = 0.13]. However, comparing the models with AQ (but without interaction) and those with only external noise showed near significant results [accuracy: χ^2^(1) = 3.44, *p* = 0.065; consistency: χ^2^(1) = 3.40, *p* = 0.065], suggesting that AQ may have a small influence, but our current experiment was not powerful enough to reveal it. Overall, there appears to be no statistically significant influence of AQ on the accuracy or consistency of participants’ reports.

#### Internal noise

**Figure 6.**
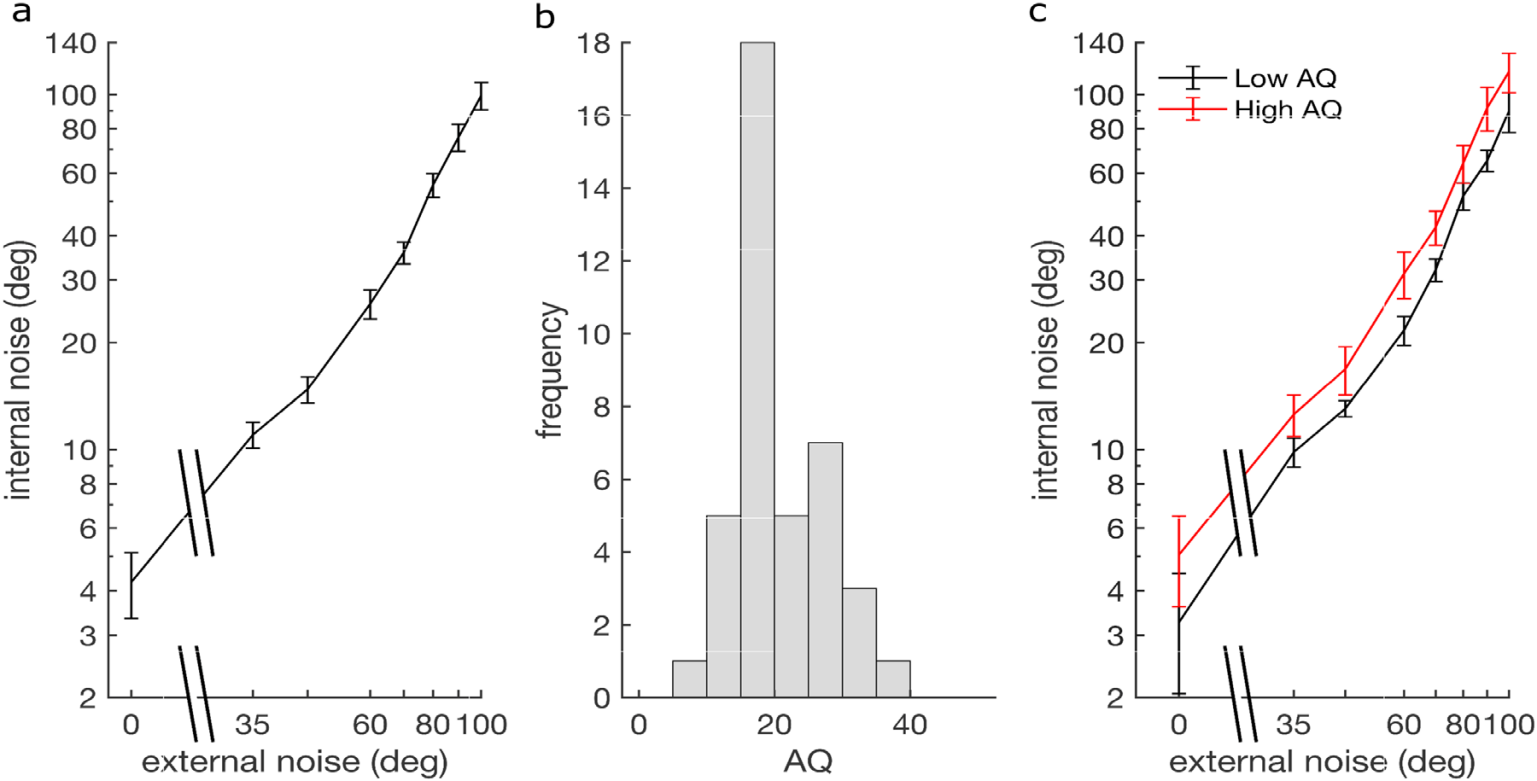
Dependence of estimated internal noise on external noise for the coarse discrimination task. (a) Average results over all participants. (b) Histogram depicting the distribution of AQ scores. (c) Internal noise after a median split for low- and high-AQ groups. Error bars indicate ±*SEM*.

Internal noise was derived from the accuracy–consistency plots (see Methods) and depended on the external noise (Figure 6a). The plot shows that internal noise values depend on external noise, a sign of the involvement of induced noise. A repeated-measures, one-way analysis of variance (ANOVA) with external noise as the factor was significant [internal noise transformed as log(σ_int_+1), *F*(2.27, 97.48) = 218.63, *p* < 0.0001, η*_p_*^2^ = 0.84, Greenhouse–Geisser corrected]. When performing median split on the AQ scores (median = 18), we obtained a group of *n* = 20 that scored AQ < 18 and a group of *n* = 20 that scored AQ > 18 (we discarded the subjects with a median score for this analysis). The mixed-design ANOVA with the factors external noise and group (low vs. high AQ) showed a significant effect of external noise [*F*(2.21, 83.78) = 202.81, *p* < 0.0001, η*_p_*^2^ = 0.84, Greenhouse–Geisser corrected], and AQ group [*F*(1, 266) = 4.29, *p* = 0.045, η*_p_*^2^ = 0.10], with internal noise increasing as external noise increased, and higher internal noise for the high AQ group. The interaction was not significant [*F*(7, 266) = 0.21, *p* = 0.98, η*_p_*^2^ = 0.005] (Figures 6b and 6c). A simple correlation over all participants between AQ and internal noise (averaged over external noise conditions) was also significant (Kendall's τ_b_ = 0.21, *p* = 0.048).

#### Additive noise

**Figure 7.**
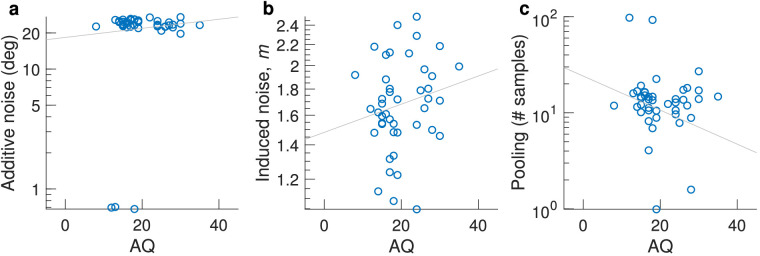
Correlations for the coarse discrimination task. (a) Additive internal noise, (b) induced noise factor *m*, and (c) motion pooling, dependent on AQ. One outlier was removed in (c).

We estimated additive and induced noise by fitting Equation 5 to individual participant data. Unfortunately, our design did not support accurate estimation of additive noise, because the task was too easy, resulting in (near) perfect performance. Our simulations indicated that internal additive noise could not accurately be estimated from this experiment, so we did not perform statistical analyses on it. However, the individual data are shown in Figure 7a.

#### Induced noise

Internal noise estimates increase with increasing external noise (Figure 6), which is a sign of the involvement of induced noise. To statistically analyze this, we fitted Equation 5 to the collective individual subject data in Figure 6, while setting *n* to 1 to reduce free parameters. This model was then compared with a model without the impact of external noise (i.e., an intercept-only model). The likelihood ratio tests whether or not the model that incorporated a dependence on external noise was better [χ^2^(1) = 190.66, *p* < 0.0001]. This emphasizes the importance of induced noise within the model.

To obtain insight into whether this involvement differs among individuals with different levels of AQ, we estimated individual levels of induced noise by fitting Equation 5. The average induced noise factor over participants was 1.70 (median, 1.67). These values did not correlate with the AQ measure (Kendall's τ_b_ = 0.09, *p* = 0.39; Bayesian correlation Kendall's τ = 0.094, *BF*_10_ = 0.29).

#### Motion pooling

We computed the motion pooling at each level of external noise for each participant using Equation 11. We then took the median value over the calculated pooling values from the external noise conditions 35°, 45°, and 60°, discarding conditions in which pooling could not be estimated. These particular external noise values were chosen based on simulations (see Methods and the Appendix). The median value was 13.73 samples, which was significantly higher than 1 (*p* < 0.0001, Wilcoxon signed-rank test) indicating that participants were responding using direction information from multiple dots. We also note that 13.73 is rather close to the square root of the number of samples present ($\sqrt{200}=14.14$), which has previously been proposed as a simple rule of thumb for estimating effective sample size in averaging tasks (Dakin, 2001). There was no correlation between the extent of motion pooling and AQ (Kendall's τ = –0.08, *p* = 0.45) (Figure 7c); one multivariate outlier was removed based on its Mahalanobis distance being >13.8155; that is, the data point deviated from the multivariate mean at a *p* < 0.001. A Bayesian correlation suggested moderate evidence for the null hypothesis (Kendall's τ = –0.083, *BF*_10_ = 0.27).

It would be intuitive to argue that motion pooling should increase the larger the external noise; therefore, we estimated internal noise at all levels of external noise using the approach outlined above. Interestingly, our behavioral data showed a very similar pattern in estimated motion pooling to the simulations (see Figure 12). This finding suggests that motion pooling is independent of the amount of external noise (as in our simulations).

## Experiment 2. Fine discrimination task

In the second experiment, we investigated how performance in a fine direction discrimination task depends on additive and induced noise, as well as on motion pooling.

### Methods

#### Participants

We recruited 37 healthy adults from the Monash University Clayton campus. All participants were proficient in English and had normal or corrected-to-normal vision, fulfilling our inclusion criteria. Participants received a monetary compensation for their participation. Participants were excluded if the internal noise model (Equation 5) fitted with an *R*^2^ < 0.5. Ten participants were excluded on this basis, leaving 27 participants in the final sample (20 females, seven males; age range, 18–32 years; *M*_age_ = 24.8; *SD*_age_ = 5.33).

#### Stimuli

Stimuli and methods were identical to the coarse discrimination task except that the mean motion direction was ±5° from vertical, and the standard deviations of the Gaussian direction distributions were 0°, 2.2°, 3.7°, 6.3°, 10.6°, 17.9°, 30.3°, and 51.2°.

#### Analyses

All analyses were as in Experiment 1, except that lookup tables for the analyses were recalculated for a signal value of 5° from vertical.

### Results

#### Influence of external noise on accuracy, consistency, and confidence

**Figure 8.**
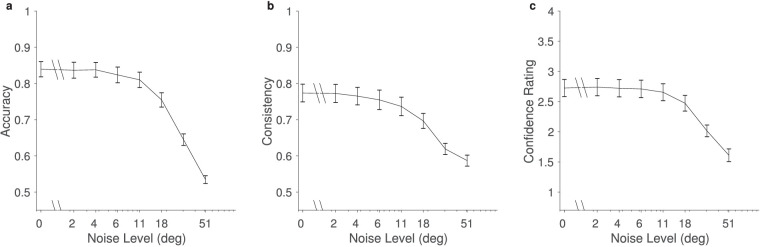
Mean behavioral performance in the fine discrimination task. (a) Accuracy, (b) consistency, and (c) confidence all decreased as external noise increased but remained significantly above chance at all noise levels. Error bars show the standard errors of the mean over participants.

As in the coarse discrimination task, the fine discrimination task showed decreases in accuracy, consistency, and confidence rating as external noise increased, reflecting increased task difficulty (Figure 8). Numerically, the accuracy and consistency were lower in people with higher AQ scores. To test the statistical significance of this finding, we constructed an unrestricted linear model with AQ, noise level, and their interaction as terms, and we compared them to models without the interaction and models without both the interaction and the AQ term. The likelihood ratio tests revealed no significant differences between unrestricted models (including AQ, noise level, and their interaction as terms) and those excluding the interaction [accuracy: χ^2^(1) = 0.068, *p* = 0.79; consistency; χ^2^(1) = 0.13, *p* = 0.72] and unrestricted models and those excluding AQ and the interaction [accuracy: χ^2^(2) = 0.07, *p* = 0.96; consistency: χ^2^(2) = 1.15, *p* = 0.56]. Comparing the models with AQ (but without the interaction) to those with only external noise showed no significant effects, either [accuracy: χ^2^(1) = 0.007, *p* = 0.93; consistency: χ^2^(1) = 1.02, *p* = 0.31]. Overall, there appears to be no influence of AQ on the accuracy or consistency in our fine motion discrimination task.

#### Internal noise

**Figure 9.**
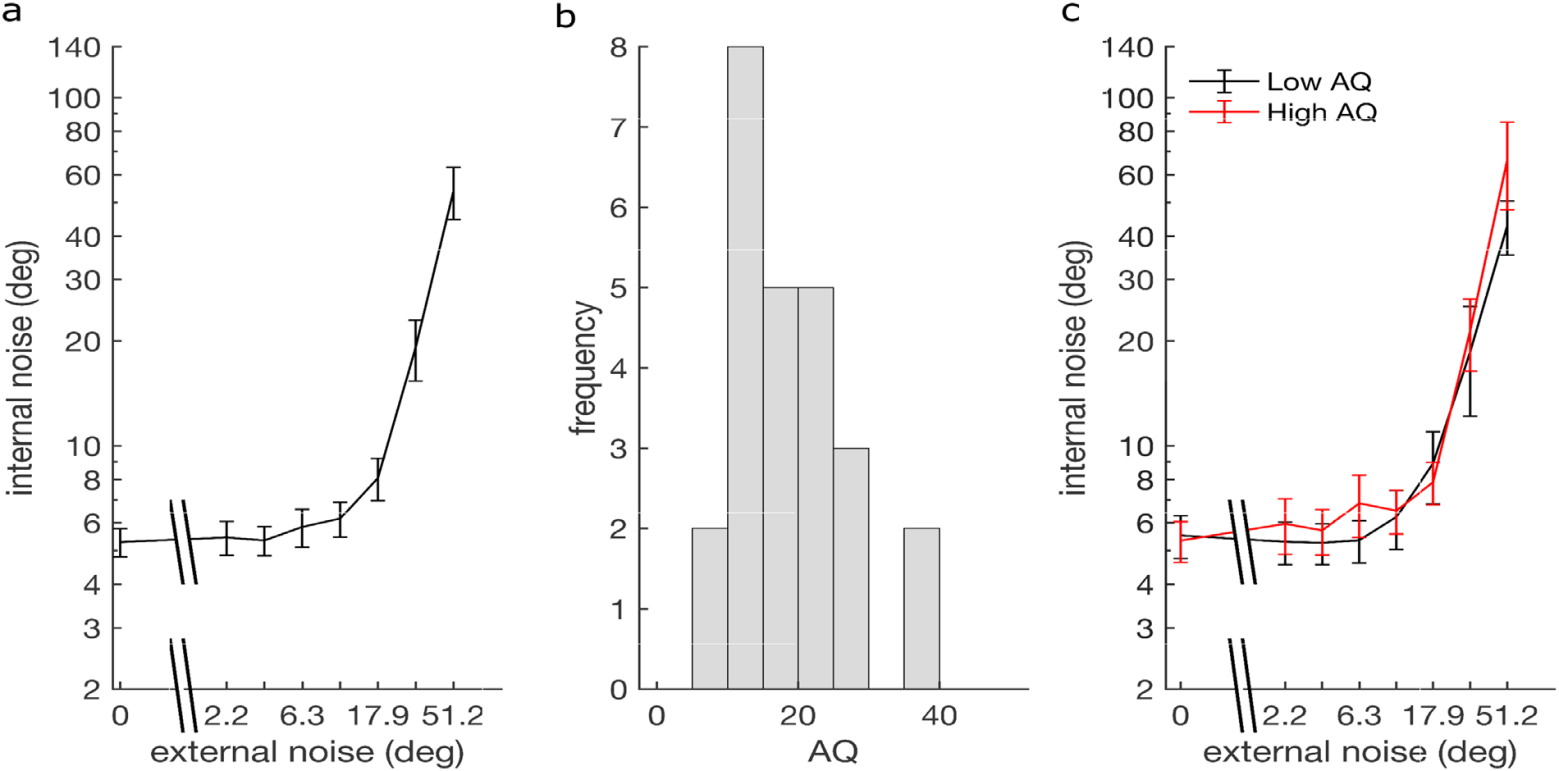
(a) Dependence of mean (±*SEM*) internal noise on external noise for the fine discrimination task, averaged over participants. (b) Histogram depicting the distribution of AQ scores. (c) Internal noise after a median split for low- and high-AQ groups. Data points that were estimated as 0 internal noise were ignored.

Internal noise in the fine discrimination task depended on the external noise (Figure 9a), which is consistent with the involvement of induced noise. A repeated-measures, one-way ANOVA with external noise as the factor was significant [internal noise transformed as log(σ_int_+1), *F*(2.92, 75.99) = 152.04, *p* < 0.0001, η*_p_*^2^ = 0.85, Greenhouse–Geisser corrected]. When performing median split on the AQ scores (median = 17), we obtained a group of *n* = 13 that scored AQ < 17 and a group of *n* = 12 that scored AQ > 17, discarding the subjects with a median score for this analysis. The mixed-design ANOVA with the factors external noise and group (low vs. high AQ) showed a significant effect of external noise [*F*(7, 161) = 131.40, *p* < 0.0001, η*_p_*^2^ = 0.85] but not AQ group [*F*(1, 161) = 0.34, *p* = 0.56, η*_p_*^2^ = 0.01]. The interaction was not significant [*F*(7, 161) = 0.21, *p* = 0.57, η*_p_*^2^ = 0.03] (Figures 9b and 9c). A simple correlation over all participants between AQ and internal noise (averaged over external noise conditions) was not significant (Kendall's τ_b_ = 0.035, *p* = 0.82).

#### Additive noise

**Figure 10.**
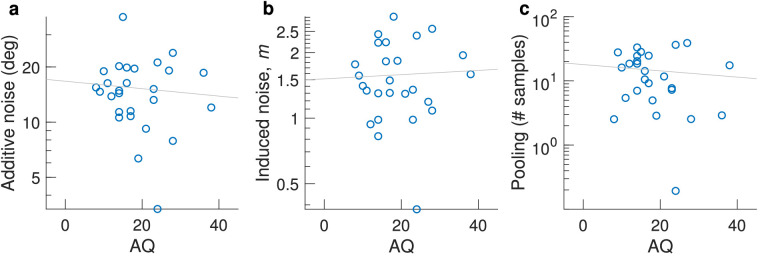
Estimates of (a) additive internal noise, (b) the induced noise factor *m*, and (c) motion pooling from the fine direction estimation task plotted against AQ.

In contrast to Experiment 1, we were able to obtain individual additive noise estimates from participants performance of the fine discrimination task. Additive noise mean was 15.24 ± 1.27 (median = 14.17; confidence interval [CI], 12.83–18.00). There was no significant correlation between our individual additive noise estimates and corresponding AQ scores (Kendall's τ = –0.07; *p* = 0.63; *BF*_10_ = 0.28) (Figure 10a).

#### Induced noise

The influence of induced noise was again shown by an increase in internal noise with increasing external noise (Figure 9). To explore this further, we fitted Equation 5 to the collective individual subject data in Figure 9 while setting *n* to 1 to reduce free parameters. This model was then compared to a model that did not incorporate this influence of external noise (i.e., an intercept-only model). The likelihood ratio tests determined whether the model with dependence on external noise was better [χ^2^(1) = 105.89; *p* < 0.0001] and showed a significant impact of induced noise.

When fitting individual subject data with Equation 5, we found that the induced noise factor, *m*, over participants was 1.72 (median, 1.49). These values were not correlated with the AQ measure (Kendall's τ_b_ = 0.019; *p* = 0.911; *BF*_10_ = 0.25; one multivariate outlier was removed) (Figure 10b).

#### Motion pooling

Mean pooling was 18.56 samples (median, 14.24), which was significantly higher than 1 (Wilcoxon signed-rank test, *p* < 0.0001). There was no significant correlation with AQ (Kendall's τ = –0.13, *p* = 0.35; one multivariate outlier was removed) (Figure 10c). A Bayesian correlation suggested that there was not sufficient data to support the absence of a correlation (*BF*_10_ = 0.39).

**Figure 12.**
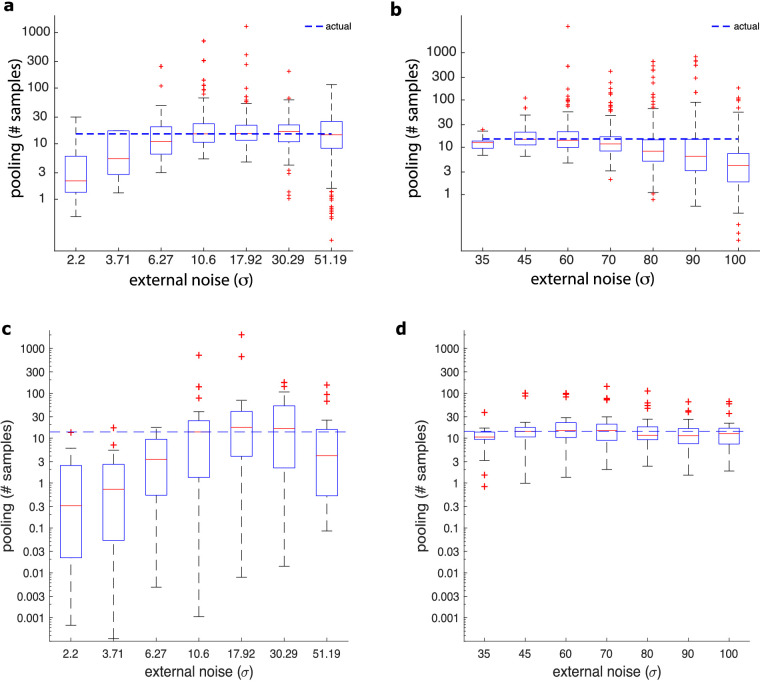
Estimated motion pooling sample size. (a) Estimates based on Equation 2 from one of the simulations. Estimates for the fine direction discrimination task were accurate for larger external noise values used in the experiment. (b) Boxplots for the coarse direction discrimination task; these estimates were accurate for the lower external noise values used in the experiment. (c, d) Experimentally obtained motion pooling estimates for fine (c) and coarse (d) discrimination tasks. The box of the boxplots contains the 25th to 75th percentiles; the whiskers show the most extreme values not considered outliers, and the red markers show the outliers.

We also estimated motion pooling at every external noise level separately. As with the coarse discrimination task, the behavioral data showed the expected pattern for a constant motion pooling, independent of external noise (see Figure 12).

#### Comparing fine and coarse discrimination tasks

From Figures 6 and 9, it appears that the internal noise is larger in the fine discrimination task than in the coarse discrimination task. We compared internal noise between the two tasks at similar external noise conditions and found that internal noise was significantly larger in two of the three comparisons (Wilcoxon rank-sum test; at σ_ext_ = 0, *Z* = 2.06 and *p* = 0.039; at fine σ_ext_ = 30.5 and coarse σ_ext_ = 35, *Z* = 1.11 and *p* = 0.27; at fine σ_ext_ = 51.2 and coarse σ_ext_ = 45, *Z* = 5.80 and *p* < 0.0001).

In terms of the dependence of internal noise on AQ, the analyses above indicated a significant effect in the coarse task but not in the fine task; however, even the fine task showed a numerically larger internal noise in the high AQ group compared with the low AQ group. In fact, over the two experiments, 14 out of the 16 noise conditions showed this overall difference. The probability of finding this difference 14 or more conditions by change is 0.0021 (binomial test), suggesting a significant effect overall.

Mean induced noise was not different between the two tasks (*Z* = 1.04; *p* = 0.30, Wilcoxon rank-sum test). Comparing the motion pooling data between coarse and fine discrimination tasks showed no significant difference (*Z* = 0.05; *p* = 0.96, Wilcoxon rank-sum test).

## Discussion

We investigated how fine and coarse motion discrimination is limited by internal noise (both additive and induced) and by pooling (or multiplicative noise), and we also examined if these limits were correlated with ASD traits in a typically developed adult population. We found evidence for higher internal noise in ASD when performing a coarse discrimination task but not in a fine discrimination task. However, in neither the coarse nor fine discrimination task were additive noise, induced noise, or motion pooling correlated with ASD traits.

### Additive noise influences on motion perception

Several studies have reported high internal noise in ASD populations compared with control groups using brain-imaging techniques such as fMRI (Dinstein et al., 2012; Haigh et al., 2015), electroencephalography (Milne, 2011; Weinger et al., 2014), and magnetoencephalography (Ishikawa, Shimegi, & Sato, 2006; Peiker et al., 2015). These results led us to anticipate a positive correlation between AQ score and additive noise which we did not observe in the fine discrimination task and which we were unable to estimate in the coarse discrimination task. Additive noise has not been previously investigated across AQ in a typically developed population, and our results suggest that additive noise does not vary with traits of ASD in the general population. These results are consistent with other research that has not found significant differences in additive noise between ASD and control groups, in both behavioral (Manning et al., 2015) and brain-imaging (Coskun et al., 2009) studies.

Aside from the lack of a link to autism traits, our results do speak to the involvement of additive noise in the perception of motion. We found considerable differences in the amount of additive internal noise between individuals. These individual differences in noise were correlated with (and, in fact, calculated from) differences in performance measures (accuracy and consistency) in a motion discrimination task. This suggests that internal additive noise can determine task performance differences among individuals, although at the moment it appears that it cannot stratify individuals along the broader ASD spectrum.

### Induced noise

We identified a strong influence of induced noise on motion direction discrimination. In motion tasks, induced noise has not been previously investigated in relation to ASD, either between ASD and control groups or across AQ score within the broader spectrum. Instead, studies have investigated internal noise without splitting it into additive and induced noise. For example, Manning et al. (2015) varied the amount of external noise in a motion discrimination task but only looked at two levels of noise. Overall, they found better performance at high noise levels for individuals with ASD relative to the control group but no difference at low noise levels. The authors tentatively attributed performance differences at high noise levels to differences in motion pooling (Manning et al., 2015), but, because motion pooling should also affect performance at low noise levels, this explanation was speculative. However, differences in induced noise provide an alternative explanation for this difference. For example, lower induced noise yields better performance at high external noise levels but not at low external noise levels (see Figure 1b), which is consistent with experimental findings in ASD (Manning et al., 2015; Manning et al., 2017). Our results do not directly support this interpretation, however, as we did not find a correlation between induced noise and AQ. Perhaps we lacked enough individuals with high AQ scores to show a clear dependence. Alternatively, large differences in induced noise may only appear when comparing a control group to a group with a clinical diagnosis of ASD.

Burgess and Colborne (1988) concluded, using a model introduced by Wilcox (1968), that induced noise could be explained by fluctuations in a decision variable if the standard deviation of the fluctuations (i.e., noise) in the decision variable is proportional to the signal-to-noise ratio (and thus to external noise when σ_ext_ >> σ_int_). This relationship can be derived as follows: variability in the decision variable (σ_dec_) adds to the variability in the response (and thus increases threshold), just like additive noise does. Thus, total noise would be $\sqrt{\sigma_{\mathrm{add}}^{2}+\sigma_{\mathrm{dec}}^{2}}$. Now, if we assume that σ_dec_ is proportional to the external noise, then total noise is $\sqrt{\sigma_{\mathrm{add}}^{2}+{\left(m\;\sigma_{\mathrm{ext}}\right)}^{2}}$, which includes the definition of induced noise.

This analysis suggests that induced noise could reflect fluctuations in a decision variable (e.g., the criterion, or an internal standard). It rests on the assumption that the decision variable is proportional to the signal-to-noise ratio, but is that warranted? Past research has shown that decision/criterion noise increases when, within an experiment over trials, a wider range of stimulus values is tested (Gravetter & Lockhead, 1973). We reason that a wider stimulus range over trials may have similar effects to a wider stimulus range within a trial (that is, stimulus noise), which implies that more stimulus noise would translate into more decision/criterion noise. This is supported by research that shows that more noisy stimuli lead to less decision confidence (which is probably related to criterion noise), even when performance (in terms of *d*′) is equated (Spence, Dux, & Arnold, 2016).

Past research in non-motion tasks (Vilidaite et al., 2017) is consistent with the interpretation that induced noise may be linked to decision noise. It was found that internal noise measured through the double-pass paradigm correlated strongly in three quite different tasks (contrast, face discrimination, and a mathematical task), and the principle component across the internal noise measures from these three tasks was correlated with AQ. These data suggest a supramodal, potentially decision-based, source of this noise.

Overall, our discussion suggests that a major difference between people with ASD and typically developing people may be in their decision process during perceptual decision-making tasks, which can be quantified using induced noise measurement, although other sources of noise will likely contribute.

### Performance levels

We show no evidence for a uniformly impoverished motion processing in individuals with increased levels of autism traits, which is consistent with the literature. We found that accuracy was generally lower in individuals with higher levels of autism traits, but this finding was not significant. In the literature, there is evidence for both increased (Foss-Feig et al., 2013; Manning et al., 2015) and decreased (e.g., Koldewyn, Whitney, & Rivera, 2010; Milne et al., 2002; Spencer & O'Brien, 2006) motion perception in ASD (see also Simmons et al., 2009).

There is a stronger case for impoverished motion perception in a particular type of global motion perception—namely, biological motion processing. Here, too, there is evidence both in favor of (e.g., Blake, Turner, Smoski, Pozdol, & Stone, 2003; Koldewyn et al., 2010; van Boxtel, Dapretto, & Lu, 2016; van Boxtel, Peng, Su, & Lu, 2017) and against (e.g., Cleary, Looney, Brady, & Fitzgerald, 2014; Cusack, Williams, & Neri, 2015; Saygin, Cook, & Blakemore, 2010) a deficit in processing, but a recent meta-analysis found that there was a small decrement in ASD versus typically developing individuals (Van der Hallen, Manning, Evers, & Wagemans, 2018). It may therefore be worthwhile to look at the influence of internal noise on biological motion (as done in van Boxtel & Lu, 2015) while focusing on the link to ASD.

### Motion pooling

Previous literature has provided both evidence for and against a difference in motion pooling in ASD versus typically developing individuals (Manning et al., 2015; Pellicano et al., 2005). We found no significant correlation between motion pooling across AQ score, and Bayesian statistics suggest that there was moderate evidence for the absence of a correlation in both discrimination tasks. As mentioned above, the increased motion pooling in ASD, reported in previous studies (Manning et al., 2015; Manning et al., 2017), is instead potentially attributable to decreased induced noise (or increased noise exclusion).

### Coarse versus fine discrimination tasks

A reduction of induced noise in individuals with more autism traits is consistent with recent reports of superior motion perception in ASD (Foss-Feig et al., 2013; Manning et al., 2015). However, several other reports show inferior performance in ASD (e.g., Koldewyn et al., 2010; Milne et al., 2002; Spencer & O'Brien, 2006), especially in motion coherence tasks (Simmons et al., 2009). These differences could be due to the large variability across individuals falling on the autism spectrum, but they could also be due to the different parameters used in the various studies. Indeed, our results support the suggestion that small parameter differences can impact the results, as we found that, although internal noise was higher for individuals with more autism traits in the coarse discrimination task, this was not the case in the fine motion discrimination.

What are the processing differences that could underlie the different findings in our fine and coarse discrimination tasks? Because the stimuli in our two experiments were nearly identical, the difference cannot be attributed to some general motion perception deficit or other commonly suggested deficits in ASD, such as a dorsal stream deficit (Grinter, Maybery, & Badcock, 2010; Spencer et al., 2000), a magnocellular dysfunction (Sutherland & Crewther, 2010), or difficulty with complex stimuli (Bertone et al., 2003; Bertone, Mottron, Jelenic, & Faubert, 2005).

Rather, our results do align with the more general notions that individuals with ASD are more “detail focused” (Happé & Frith, 2006; Mottron, Dawson, Soulieres, Hubert, & Burack, 2006) and only show increased internal noise in the coarse discrimination task. An alternative, not mutually exclusive, account is that different “decoding rules,” or decision rules, are used for fine and coarse discrimination tasks (Jazayeri & Movshon, 2007), and that they are differently affected by noise and autism traits. This suggestion is supported by our comparison of internal noise values between the two tasks. We found that internal noise was larger in the fine discrimination task. Our data suggest that the type of task influences the amount of internal noise, even with near identical stimuli. Caution is warranted, however, as we used different participants in both experiments.

## Conclusions

This research is the first to attempt to relate AQ scores to estimates of additive and induced noise limits on motion direction discrimination. We have shown the influences of additive and induced noise, as well as motion pooling, on motion perception. Our results are suggestive of an increase in internal noise in individuals with more autism traits in some conditions, but the individual noise measures of additive noise, motion pooling, and induced noise do not correlate with ASD traits. This suggests that a combination of these three (and potentially other) factors, increases internal noise and that there is no individual type of noise that is solely responsible for the overall increase in internal noise.

We ascribe induced noise to variability in decision making and argue that this could provide an alternative explanation of past results indicating superior motion averaging in ASD. The involvement of induced noise in motion perception is very relevant to ASD research, because induced noise affects the perception of suprathreshold stimuli, as opposed to additive noise which mostly affects perithreshold perception. This suggests internal and specifically induced noise as an explanation for (perceptual) atypicalities in ASD, including hyper- and hyposensitivity.

## Figures and Tables

**Figure 11.**
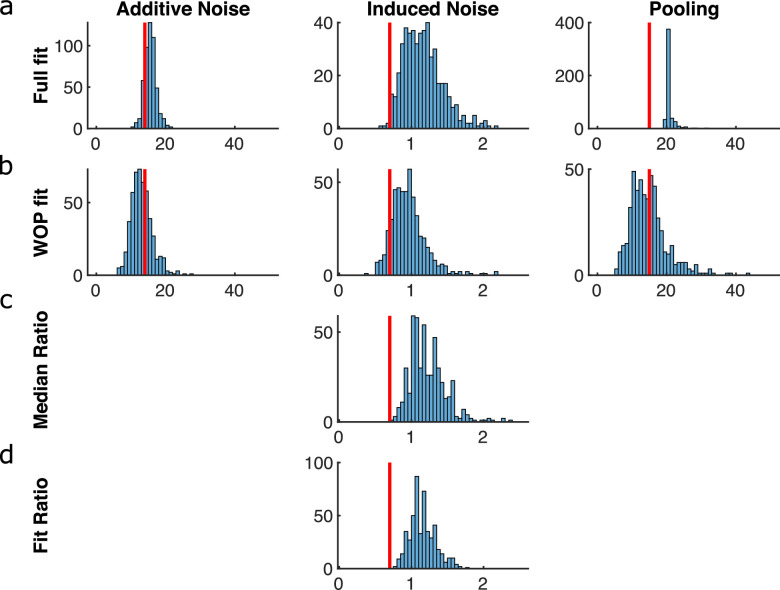
Simulation results for different fitting procedures for the fine direction discrimination experiment. Results show histograms of estimated model parameters from 500 simulated runs. The red lines indicate the true values. (a) Full fit in which additive noise, induced noise, and motion pooling were all individually fit. (b) Fit without pooling (WOP) in which additive noise and induced noise were fitted, but the pooling parameter was estimated directly from the data. (c) Estimated induced noise, the median induced noise value for the five largest external noise conditions. (d) Estimated induced noise taken from a single fit through the five largest external noise conditions.

## Footnotes

1 Induced noise may also be interpreted in terms of noise exclusion (i.e., the ability to have external noise not influence internal noise). In this article, we do not distinguish between these different processes. Another type of noise mentioned in the literature is multiplicative noise, which scales both signal and noise. In our paradigm, this behavior is interpreted as motion pooling.

2 We use the simplest descriptive model that describes our data. It is possible to construct more complex hierarchal models. But, even if they are better descriptions of the different components of noise, those models are underdetermined by our current dataset. This does mean, however, that the exact values of our estimates from the simple model are probably not reflective of any single source of noise in the brain.

## Appendix

We performed a simulation study to investigate which approach best estimates the different sources of internal noise and motion pooling. We used 100 double-pass trials (200 trials total), just as for our experimental data. We set additive noise, induced noise, and motion pooling to reasonable values. If the final activity (that is, signal plus noise) was larger than zero, a “right” response was recorded; otherwise, a “left” response was recorded. Accuracy and consistency values were calculated and then fitted with the same procedures as in the experiment. This resulted in internal noise estimates at each external noise level. Using the following simulations, we aimed to determine which method was best able to extract the original additive noise, induced noise, and motion pooling values. The code for these simulations can be found at https://osf.io/4gdkt/.

The first approach was to fit one function to the whole internal noise versus external noise curve (like our data in Figures 6 and 9). According to the definition of induced noise (Burgess & Colborne, 1988) total internal noise is equal to $\sqrt{{\sigma_{\mathrm{add}}^{2}+\left(m\;\sigma_{\mathrm{ext}}\right)}^{2}}$. This definition, however, does not include the possible effect of motion pooling, which we observed in our data, so we used the following function (see Equation 5):

$$\;\sigma_{\mathrm{int}}\left(\sigma_{\mathrm{ext}}\right)=\sqrt{\frac{\sigma_{\mathrm{add}}^{2}+\;(m\;{\sigma_{\mathrm{ext}})}^{2}}{n}}$$

We tested several ways of fitting this function. First, we let all parameters (σ_add_, *m*, and *n*) vary freely. Second, we estimated *n* from our data using Equation 11 and estimated only σ_add_ and *m*. The third and fourth approaches were based on the following approximation: as σ_ext_ becomes large, σ_int_ approaches *m*σ_ext_, which means that *m* = σ_int_/σ_ext_, or the σ_int_/σ_ext_ ratio that we measure with the double-pass paradigm. This method ignores the influence of motion pooling, which can be included and would lead to the following estimate: $m=\;\sqrt{n}*\;\sigma_{\mathrm{int}}/\sigma_{\mathrm{ext}}$. We found, however, that this latter estimate severely overestimates *m*, and we do not discuss it here. The third method derived σ_int_/σ_ext_ by fitting σ_int_/σ_ext_ curves through each of the data points from the largest five external noise values and taking the median value of these fits. The fourth method derived σ_int_/σ_ext_, fitting one σ_int_/σ_ext_ curve (Figure 4) through the data from the largest five external noise values only, and this ratio was taken as the induced noise factor *m*.

Each simulation performed 500 runs, resulting in a distribution of extracted values for additive noise, induced noise, and motion pooling. We then performed different simulations and checked which approach overall appeared to give the best estimates of the inputted additive noise, induced noise, and motion pooling.

Figure 11 shows the results for one of these simulations for the small-angle experiment. It shows (as most simulations did) that the best approach was to estimate pooling from the data and fit additive noise and induced noise (Figure 11b; the WOP fit, which is the fit without motion pooling). The approach in which all parameters were fitted was very dependent on initial conditions (this was because many parameter combinations led to virtually identical fits). The other two approaches overestimated induced noise (and did not provide estimates for additive noise and pooling). The simulations for the large-angle data showed similar findings but also indicate that additive noise could not be estimated very well for this experiment (not shown). We therefore decided not to do statistics on the additive noise measures for the large-angle experiment.

These simulations also indicated that motion pooling could be estimated from raw data using Equation 2, but only for higher external noise values for the small angle experiment (Figure 12a) and for the lower external noise values for the large-angle experiment (Figure 12b). In these simulations, we used a fixed level of motion pooling (dashed horizontal line), but systematic underestimation occurred at various external noise levels. Therefore, we decided to use only conditions with external noise being 35°, 45°, or 60° for our coarse discrimination task and 3.71° to 51.19° for the fine discrimination task.

Interestingly, when we used the motion pooling estimation approach on all our experimental data (Figures 12c and 12d) the same pattern as in our simulation appeared, strongly suggesting that motion pooling is constant across external noise levels.
